# Gametocyte-Specific Factor 1 Is Essential for Male Fertility and Transposon Homeostasis in *Plutella xylostella*

**DOI:** 10.3390/insects17080846

**Published:** 2026-08-14

**Authors:** Jianying Liao, Muhammad Asad, Yanpeng Chang, Beihua Qin, Chengbin Liu, Xiping Du, Jinzhi Chen, Guang Yang

**Affiliations:** 1 State Key Laboratory of Agricultural and Forestry Biosecurity, Institute of Applied Ecology, Fujian Agriculture and Forestry University, Fuzhou 350002, China; 2 Joint International Research Laboratory of Ecological Pest Control, Ministry of Education, Fuzhou 350002, China; 3 Ministerial and Provincial Joint Innovation Centre for Safety Production of Cross-Strait Crops, Fujian Agriculture and Forestry University, Fuzhou 350002, China; 4 Key Laboratory of Integrated Pest Management for Fujian-Taiwan Crops, Ministry of Agriculture, Fuzhou 350002, China; 5 Key Laboratory of Green Pest Control, Fujian Province University, Fuzhou 350002, China; 6 College of Ocean Food and Biological Engineering, Jimei University, Xiamen 361021, China; 7 Southern Zhejiang Key Laboratory of Crop Breeding, Wenzhou Vocational College of Science and Technology (Wenzhou Academy of Agricultural Sciences), Wenzhou 325006, China

**Keywords:** Gtsf1, CRISPR/Cas9, male fertility, piRNA pathway, *Plutella xylostella*

## Abstract

Gametocyte-specific factor 1 (Gtsf1) plays a pivotal role in the gonadal development by transposon silencing via the piRNA pathway in *Drosophila melanogaster* and *Bombyx mori*, and is implicated in the female sex determination in *B. mori*. However, its function remains elusive in other insect species, including *Plutella xylostella*, a highly destructive pest in cruciferous crops. In this study, we characterized PxGtsf1, encoding a 27.84 kDa protein containing two conserved U11-48K-like CHHC zinc-finger domains. *PxGtsf1* was predominantly expressed in male pupae, males and testes. Using CRISPR/Cas9 technique, we successfully generated a homozygous *PxGtsf1*-knockout strain (△PxGtsf1) with 11 bp deletion. △PxGtsf1 females exhibited normal morphology in terms of external genitalia, body weight, ovariole morphology, ovariole length, oocytes number, and internal ovarian structure, and maintained the expression levels of genes related to female sex determination and fertility. In contrast, △PxGtsf1 males displayed reduced testis size and enlarged eupyrene sperm bundles, whereas apyrene sperm bundles appeared normal. Wild-type (WT) or △PxGtsf1 females mated with △PxGtsf1 males had significantly smaller bursa copulatrix distention area compared to those mated with WT males. Furthermore, WT or △PxGtsf1 females mated with △PxGtsf1 males produced significantly fewer eggs with lower hatching rates than those mated with WT males. Co-immunoprecipitation assays revealed that PxGtsf1 specifically interacts with PxPiwi. Small RNA sequencing further showed that *PxGtsf1* knockout resulted in transposon dysregulation in the testes without affecting overall piRNA abundance, suggesting that PxGtsf1 functions in piRNA-mediated transposon regulation rather than global piRNA biogenesis. *PxGtsf1* is first reported to be specifically required for male fertility in Lepidoptera insects but is dispensable for female sex determination., PxGtsf1 regulates transposon homeostasis without affecting global piRNA biogenesis. These findings provide new insights into the diverse functions of Gtsf1 in Lepidopteran insects.

## Simple Summary

The Gametocyte-specific factor 1 (Gtsf1) gene is known to modulate gonadal development and sex determination via the piRNA pathway in limited insect species, while its biological function in the destructive cruciferous pest *Plutella xylostella* remains unknown. Here, we functionally characterized *PxGtsf1* in diamondback moths using CRISPR/Cas9-mediated gene knockout. *PxGtsf1* was specifically enriched in male pupae and testes, and its deletion caused defective male testicular morphology and sperm structure, further reducing mating fecundity and egg hatching efficiency. No obvious abnormalities were observed in the female reproductive system and sex determination process after *PxGtsf1* knockout. Molecular verification confirmed that PxGtsf1 interacts with PxPiwi to maintain testicular transposon homeostasis, without influencing piRNA biosynthesis. This study clarifies a novel sex-specific function of Gtsf1 in regulating lepidopteran male fertility, which updates the understanding of insect Gtsf1 functional diversity.

## 1. Introduction

Gametocyte-specific factor 1 (Gtsf1), also known as Asterix in *Drosophila*, is a highly conserved, germline specific small protein characterized by two tandem CHHC type zinc finger domains at its N terminus and an intrinsically disordered C terminal region [1,2]. Since its initial characterization, Gtsf1 has been established as an indispensable factor for germline development and genomic stability maintenance across diverse metazoan models. Its core function is intimately linked to the PIWI interacting RNA (piRNA) pathway, a critical defense system that silences transposable elements and safeguards genome integrity in the animal germline [3,4].

Strikingly, Gtsf1 has exhibited remarkable evolutionary plasticity in terms of its functional roles across different species. Although it serves as a core cofactor of the piRNA pathway, the precise molecular nodes at which it acts, its mechanistic details, and the biological processes it participates in display clear species-specific features.

In mammals, mouse (*Mus musculus*) Gtsf1 is absolutely required for male fertility. It functions as a critical cofactor for PIWI proteins and is essential for secondary piRNA biogenesis, its loss leads to defective retrotransposon silencing and male sterility [5,6]. Recently, human genetic studies have confirmed that biallelic mutations in the *Gtsf1* gene cause male infertility, with patients exhibiting impaired piRNA biogenesis and transposon depression [7]. PtGtsf1 from the unicellular protozoan *Paramecium tetraurelia* also conservatively binds PIWI proteins and solely governs transposon-associated DNA elimination during meiosis [8,9]. The role of Gtsf1 in *Caenorhabditis elegans* is even more unusual; it does not participate in transposon silencing at all, nor does it associate with PIWI proteins, instead, it forms a complex with the RNA dependent RNA polymerase RRF 3 and is involved in the synthesis of another class of small RNAs, the 26G interfering RNAs, and its mutants remain fully fertile [10]. In insects, the functions of Gtsf1 are even more diversified. In *Drosophila melanogaster*, DmGtsf1 is essential for female fecundity; it localizes predominantly to the nucleus and collaborates with the Piwi protein to mediate transcriptional transposon silencing, without being involved in piRNA biosynthesis [11,12]. In striking contrast, silkworm (*Bombyx mori*) BmGtsf1 not only participates in gametogenesis of both sexes but also possesses a unique, indispensable function in female sex determination [13]. Silkworm female sex determination relies on the W chromosome derived *Feminizer* (*Fem*) piRNA [14]; BmGtsf1 interacts with BmSIWI and is indispensable for *Fem* piRNA biogenesis and for the silencing of the masculinization gene *BmMasc* [15]. Mutations in BmGtsf1 cause partial sex reversal in genotypic females (WZ) [13].

Collectively, although Gtsf1 is a conserved component of the piRNA pathway, its functions have undergone substantial divergence from unicellular protozoan to mammal. These findings together paint a complex picture of functional diversification and evolutionary plasticity of Gtsf1. Nevertheless, it remains unclear whether the functions of Gtsf1 in Lepidoptera are universally conserved, and in particular, whether its role in sex determination is a silkworm specific acquisition. The diamondback moth (*Plutella xylostella*), as another important lepidopteran model, possesses a sex determination mechanism that involves the *Masc* gene [16], but its upstream signaling does not fully align with the *Fem* piRNA system of the silkworm [17]. In *P. xylostella*, the piRNA pathway is mainly mediated by the PIWI family proteins, including PxPiwi and PxAgo3. However, the functional relationship between Gtsf1 and these PIWI proteins remains largely unexplored. Therefore, investigating the functional role of Gtsf1 in *P. xylostella* will not only advance our understanding of the functional evolution of this gene across insects, but may also provide new targets for genetic pest management in lepidopteran pests.

## 2. Materials and Methods

### 2.1. Insect Strains and Rearing

The G88 strain of *P. xylostella* was maintained in the Institute of Application Ecology, Fujian Agriculture and Forestry University, Fuzhou, China. Larvae were reared on a standardized artificial diet as previously described [18], while adult moths were provided with a 10% honey solution following established protocol [19].

### 2.2. RNA Extraction and cDNA Synthesis

Tissues including fat body, head, midgut, Malpighian tubule, silk gland, integument, ovary and testis, and individuals of different developmental stages (egg, larva, pupa, and adult), were collected for sample preparation. Total RNA was extracted from all samples using the RNA Simple Total RNA Kit (TIANGEN Biotech, Beijing, China). RNA quality was verified via 1% agarose gel electrophoresis, and 1 μg of high-quality total RNA was reverse-transcribed into cDNA using the Reverse Transcription Kit (Vazyme Biotech, Nanjing, China).

### 2.3. PCR Amplification and Cloning of PxGtsf1

The full-length open reading frame (ORF) of *PxGtsf1* was retrieved from the genomic database of *P. xylostella* [20] and gene-specific primers (sequences listed in Table S1) were designed using Primer 5 software. PCR amplification was performed using Phanta Max Super-Fidelity DNA Polymerase (Vazyme Biotech, Nanjing, China). The resulting PCR product was purified, ligated into the pEASY Simple Blunt Cloning Vector (Yeasen Biotech, Beijing, China) following the manufacturer’s protocol, and verified by Sanger sequencing.

### 2.4. In Silico Analysis of PxGtsf1

Amino acid sequences of Gtsf1 orthologs from Lepidoptera, Coleoptera, Orthoptera, Diptera, and Hemiptera were retrieved from the National Center for Biotechnology Information (NCBI) database. Multiple sequence alignment was performed using MEGA X software, and the resulting alignment was visualized with ESPript 3.2 (https://espript.ibcp.fr/ESPript/cgi-bin/ESPript.cgi; accessed on 15 March 2024) [21]. Conserved domain architecture was analyzed via the NCBI Batch CD-Search tool (https://www.ncbi.nlm.nih.gov/Structure/bwrpsb/bwrpsb.cgi; accessed on 15 March 2024). The neighbor-joining (NJ) phylogenetic tree was constructed in MEGA X based on amino acid sequences [18]. The Poisson model was used to calculate pairwise amino acid distances, with uniform substitution rates among sites and homogeneous evolutionary patterns across lineages. Gaps and missing data were treated with complete deletion. Node support was evaluated using the bootstrap method with 1000 replicates. Branch lengths correspond to amino acid genetic distances. The phylogenetic tree was visualized using the ChiPlot online platform (https://chiplot.online/; accessed on 15 March 2024).

### 2.5. Quantitative Real-Time PCR (qRT-PCR)

Quantitative real-time PCR (qRT-PCR) was performed using the SYBR Green qPCR Master Mix (Vazyme Biotech, Nanjing, China) to quantify the expression levels of target genes. Gene expression levels were normalized to the reference gene *PxRPL32* (*ribosomal protein L32*). All experiments were conducted with three independent biological replicates, each including three technical replicates. The sequences of all qRT-PCR primers are provided in Table S1.

### 2.6. Design and In Vitro Synthesis of sgRNA

Small guide RNA (sgRNA) target sequences were designed following the 5′-GG-(N)_18_-NGG-3′ principle using the CRISPR RGEN Tools online platform (https://www.atum.bio/eCommerce/cas9/input; accessed on 15 May 2024) [18]. Potential off-target effects and sgRNA mismatches were predicted and evaluated with the Cas9 Off-Finder tool (http://www.rgenome.net/cas-offinder/; accessed on 15 May 2024) [18]. Two oligonucleotides targeting the sgRNA scaffold were designed (Table S1) and template was amplified via PCR using Phanta Max Super-Fidelity DNA Polymerase (Vazyme Biotech, Nanjing, China). The resulting PCR product was purified with the Universal DNA Purification Kit (TIANGEN Biotech, Beijing, China), and subsequently used as a template for in vitro transcription of sgRNA with the HiScribe™ T7 Quick High Yield RNA Synthesis Kit (New England Biolabs, Ipswich, MA, USA) according to the manufacturer’s protocol. The transcribed sgRNA was further purified using RNA Extraction Reagent (Solarbio Life Sciences, Beijing, China).

### 2.7. Embryo Microinjection

GenCrispr Cas9-NLS nuclease protein (200 ng/μL; GenScript Biotech Corporation, Nanjing, China) was mixed with sgRNA (100 ng/μL) at a molar ratio of 1:2, and the mixture was incubated at 37 °C for 20 min to allow the formation of ribonucleoprotein (RNP) complexes. The resulting RNP complexes were microinjected into freshly laid eggs at the preblastodermal stage, collected within 15 min after oviposition. Microinjections were performed using an Olympus SZX16 microinjection system (Olympus Corporation, Tokyo, Japan), and all injections were completed within 30 min after egg collection. Injected embryos were incubated in complete darkness at 25 ± 1 °C with 60% ± 10% relative humidity until hatching.

### 2.8. Mutagenesis Analysis

Genomic DNA was extracted from somatic mutants using the TIANamp Genomic DNA Kit (TIANGEN Biotech, Beijing, China). Approximately 100 ng of purified genomic DNA was used as template for PCR amplification with gene-specific primers PxGtsf1-seq-F and PxGtsf1-seq-R (Table S1). The resulting amplicons were bidirectionally sequenced to confirm the mutation status of *PxGtsf1*.

### 2.9. Immunofluorescence Analysis

Testes from newly eclosed male adults were dissected in ice-cold phosphate-buffered saline (PBS, pH 7.4) to isolate intact testicular tissues. The testes were gently ruptured with fine forceps to release mature sperm bundles, which were fixed in 4% (*w*/*v*) paraformaldehyde solution for 30 min at room temperature. After three consecutive washes with 1× Tris-buffered saline with Tween 20 (TBST, pH 7.6) for 5 min, each sample was permeabilized with 0.1% Triton X-100 in 1× TBST for 30 min at room temperature, followed by another three washes with 1× TBST.

Sperm bundles were then blocked with 3% blocking solution (1.5 g BSA in 50 mL 1× TBST) for 1 h at room temperature and washed three times with 1× TBST. The rabbit polyclonal antibody against PxGtsf1, PxAgo3 and PxPiwi was custom-produced by GenScript (Nanjing, China) using a KLH-conjugated synthetic peptide as immunogen and purified via antigen affinity chromatography. Samples were incubated with primary antibody diluted 1:200 in blocking solution at room temperature for 4 h, followed by three washes with 1× TBST. Subsequently, samples were incubated with the Alexa Fluor 594-conjugated secondary antibody (1:200 dilution in blocking solution, Thermo Fisher Scientific, Waltham, MA, USA) in the dark for 2 h at room temperature and washed five times with 1× TBST. Finally, samples were counterstained with the DAPI Fluoromount-G™ (Em = 455 nm; Yeasen, Shanghai, China) for 10 min and examined by using a Leica SP8 confocal laser-scanning microscope (Leica, Wetzlar, Germany).

### 2.10. Reproductive Organ Analysis

Virgin females and males from both the mutant and WT strains were dissected in ice-cold phosphate-buffered saline (PBS, pH 7.4) to isolate reproductive organs. Ovary or testis morphology was imaged, and the length of ovary or cross-sectional area of each testis was quantified using a VHX-2000C digital microscope (Keyence Corporation, Osaka, Japan).

WT virgin females were mated with either WT or mutant males for 40 min, then dissected in ice-cold PBS to collect the bursa copulatrix. The morphological changes in the bursa copulatrix were analyzed, and its area was measured using the same VHX-2000C digital microscope and ImageJ (v 1.53t99) software as described above.

### 2.11. Fluorescent Staining

Newly eclosed females or males were dissected in ice-cold phosphate-buffered saline (PBS, pH 7.4) to isolate the ovary or testes. Isolated testes were gently ruptured with fine forceps to release mature sperm bundles. Ovary or sperm bundles were immediately fixed in 4% (*w*/*v*) paraformaldehyde solution for 15 min at room temperature. After three washes with PBS (5 min each), ovary or sperm bundles were stained with DAPI Fluoromount-G™ (Yeasen Biotechnology, Shanghai, China) for 10 min in the dark. Fluorescence signals were visualized and captured using a Leica SP8 confocal laser-scanning microscope (Leica Microsystems, Wetzlar, Germany).

### 2.12. Bioassay for Fecundity

One-day-old virgin adults from both the mutant and WT strains were individually paired in transparent mating chambers. Each pair was provided with a 10% honey solution as a nutritional supplement. Egg-laying substrates were replaced and collected every 24 h over a 3-day period. Total egg production was recorded daily, and egg hatchability was calculated after a 72 h incubation period under standard rearing conditions.

### 2.13. Mating Behavior Analysis

Newly eclosed mutant males and females were separated and either crossed with newly emerged WT adults of the opposite sex or sib-mated in plastic cups. The response time and the mating time were recorded for each pair. Twenty biological replicates were conducted for mating time and response time.

### 2.14. Co-Immunoprecipitation Assay

The full-length coding sequence (CDS) of *PxGtsf1* was fused in-frame to the N-terminus of red fluorescent protein (RFP), while the complete CDS of *PxPiwi* and *PxAgo3* were fused to the N-terminus of green fluorescent protein (GFP), respectively. All recombinant plasmids were purified using the EndoFree Mini Plasmid Kit (TIANGEN Biotech, Beijing, China) to ensure endotoxin-free preparations. The constructs were co-transfected into Sf9 cells using Lipofectamine™ 3000 Transfection Reagent (Thermo Fisher Scientific, Waltham, MA, USA) following the manufacturer’s standard protocol.

Cells were harvested 48 h post transfection, and co-immunoprecipitation (Co-IP) assays were performed using the Biolinkedin^®^ GFP-Tag IP/Co-IP Kit (Biolinkedin Biotechnology, Shanghai, China) according to the manufacturer’s instructions. Immunoprecipitated proteins were separated by sodium dodecyl sulfate–polyacrylamide gel electrophoresis (SDS–PAGE) using the Color PAGE Gel Rapid Preparation Kit (Epizyme Biotech, Shanghai, China). Separated proteins were transferred to polyvinylidene fluoride (PVDF) membranes, which were probed with rabbit anti-RFP polyclonal antibody (1:5000 dilution) and mouse anti-GFP polyclonal antibody (1:5000 dilution). Horseradish peroxidase (HRP)-conjugated goat anti-rabbit IgG and goat anti-mouse IgG (both at 1:10,000 dilution) were used as secondary antibodies. Protein signals were detected using the BeyoECL Plus kit (Beyotime Biotechnology, Shanghai, China) according to the manufacturer’s protocol.

### 2.15. Small RNA Sequencing

Testes from WT and mutant males were dissected then submitted to the Applied Protein Technology (Shanghai, China) for small RNA sequencing.

Raw small RNA reads were quality-trimmed to remove adapters and low-quality sequences, and reads shorter than 18 nt were discarded. Contaminant sequences (rRNA, tRNA, snRNA, snoRNA, and known miRNAs) were filtered out, and clean unique reads were collapsed and labeled with sample origin and abundance information for weighted quantification. A comprehensive TE reference library for *P. xylostella* was constructed by merging Repbase-known repeats [22] and de novo predicted TE consensus sequences generated by RepeatModeler2 [23] and annotated via RepeatMasker [6].

Clean reads were aligned to the integrated TE library using Bowtie 1.1.2, permitting zero mismatches and retaining up to 100 optimal alignments per read [6]. Multi-mapping reads were preserved to avoid systematic loss of TE-derived piRNAs, which typically map to multiple repetitive loci.

To eliminate overcounting bias, multi-mapped reads were fractionally weighted according to total read abundance/number of aligned loci. Candidate piRNAs were defined as 24–30 nt TE-mapped reads. Differential expression of TE-derived piRNAs between WT and ΔPxGtsf1 groups was analyzed using log_2_-transformed reads per million (RPM) values. Unpaired Student’s *t*-test was applied, and significantly altered TE-piRNAs were defined by thresholds of |log_2_(fold change) | ≥ 1 and FDR < 0.05.

All TE-derived piRNAs were profiled for two canonical ping-pong cycle hallmarks: 5′ terminal uridine bias (1U) of antisense piRNAs and 10th-position adenine bias (10A) of paired sense piRNAs. For ping-pong pattern detection, sense–antisense piRNA pairs mapped to identical TE loci were analyzed. The 5′–5′ offset distance was calculated as the antisense piRNA 5′ coordinate minus the sense piRNA 5′ coordinate, corresponding to an overlap length of *d* + 1. Weighted pairing counts were quantified for overlaps ranging from 1 nt to 30 nt to characterize global ping-pong distribution. Ping-pong enrichment was quantitatively evaluated using a Z-score for the canonical 10 nt piRNA pair overlap [6].

Reads mapped to RepeatMasker-defined TE regions were collected and normalized to RPM to quantify TE transcriptional levels. TEs with thresholds of |log_2_(fold change) | ≥ 1 and FDR < 0.05 were identified as DE-TEs. Genomic copy number correction was performed to filter TEs with differential abundance caused by copy number variation. All downstream visualization was generated using RPM values.

### 2.16. Statistics Analysis

To assess the significant differences in PxGtsf1 expressions among various stages and tissues, as well as in male fertility and mating behavior including egg laying, hatchability, response time and mating time, one-way ANOVA followed by Tukey’s test at a significance level of *p* < 0.05 was employed. The independent *t*-test at *p* < 0.05 was conducted to determine significant differences in pupal weight, ovariole length, oocyte number, female fertility, testis size, size of bursa copulatrix and unique reads. All statistical analyses were conducted using SPSS Statistics 22.0 (IBM Corporation, Armonk, NY, USA). Graphical illustrations were generated using GraphPad Prism 10.0.

## 3. Results

### 3.1. Sequence Characterization and Expression Profile of PxGtsf1

**Figure 1:**
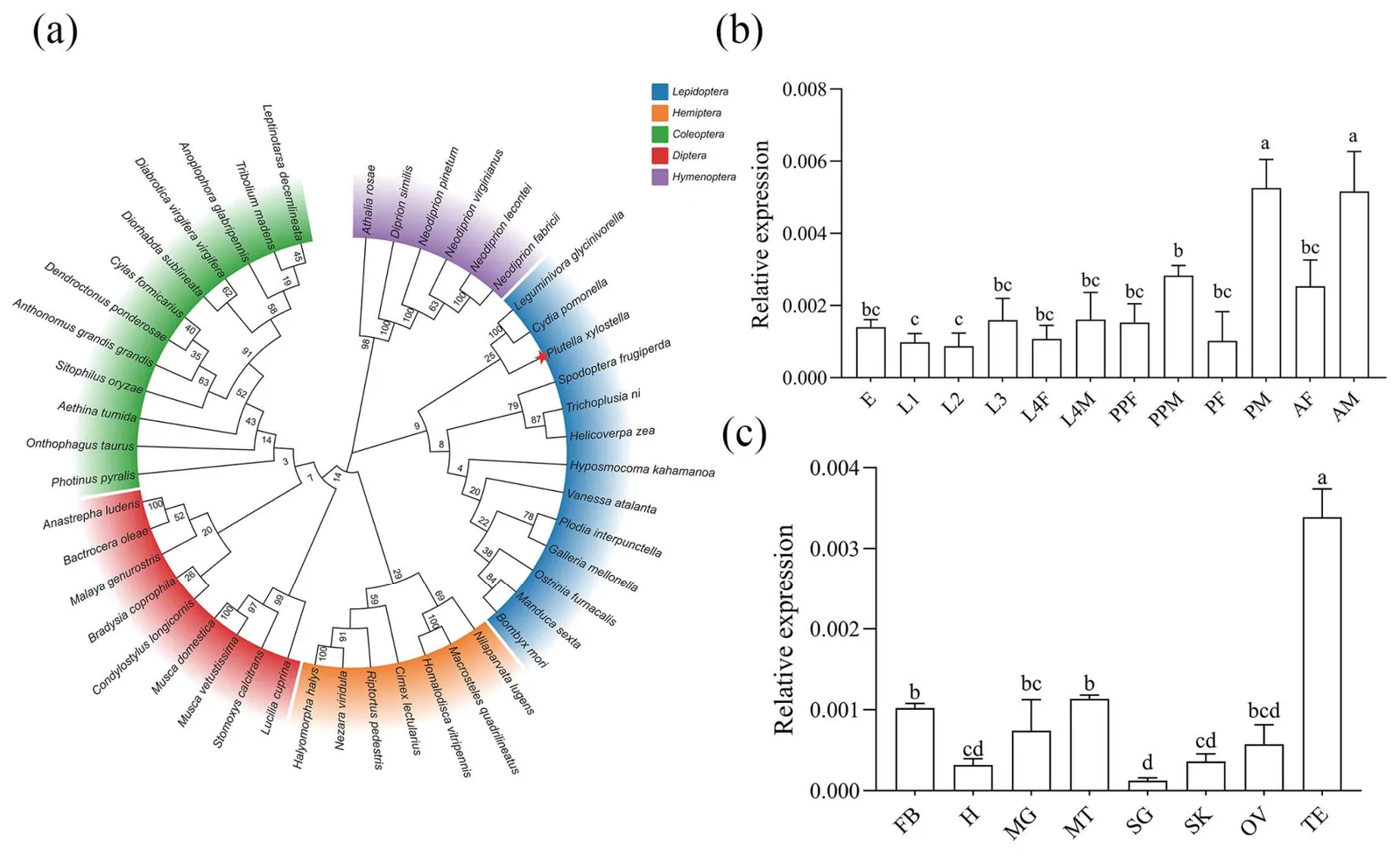
Phylogenetic analysis of insect Gtsf1 proteins and expression profiling of PxGtsf1 in *P. xylostella*. (**a**) Phylogenetic tree of insect Gtsf1 proteins. The tree was constructed by using the neighbor-joining method with sequences from 47 insect species representing five orders. Species were clustered into five groups according to their respective orders, with each group highlighted in a different color. The red star marks *P. xylostella.* Bootstrap values are shown at the corresponding branch nodes. (**b**) Relative expression levels of *PxGtsf1* gene at different developmental stages. E, egg; L1, 1st-instar larva; L2, 2nd-instar larva; L3, 3rd-instar larva; L4F, 4th-instar female larva; L4M, 4th-instar male larva; PPF, female prepupa; PPM, male prepupa; PF, female pupa; PM, male pupa; AF, female adult; AM, male adult. (**c**) Relative expression levels of *PxGtsf1* gene in different tissues. FB, fat body; H, head; MG, midgut; MT, malpighian tubule; SG, silk gland; SK, skin; OV, ovary; TE, testis. Bars represent the mean ± standard error of the mean (SE), and different letters above the bars indicate statistically significant differences (*p* < 0.05).

The *Gtsf1* gene sequence of *B. mori* (GenBank accession no. XM_004922139) was retrieved from NCBI and used as a query to perform a BLAST (https://blast.ncbi.nlm.nih.gov/Blast.cgi?PAGE_TYPE=BlastSearch; accessed on 23 January 2026) search against the *P. xylostella* genome. A highly homologous sequence with the ID Px005061 was identified. The sequence contained an ORF of 444 bp, encoding 148-amino-acid protein. Similar to Gtsf1 orthologs from other lepidopteran insects, including the silkworm (*B. mori*), soybean pod borer (*Leguminivora glycinivorella*), fall armyworm (*Spodoptera frugiperda*), cabbage looper (*Trichoplusia ni*), Indian meal moth (*Plodia interpunctella*), and Asian corn borer (*Ostrinia furnacalis*), PxGtsf1 protein contained two ZF-U11-48K domains (Figure S1). Multiple sequence alignment of Gtsf1 proteins from these six lepidopteran species revealed that PxGtsf1 shared 49% identity with BmGtsf1, 58% with LgGtsf1, 40% with SfGtsf1, 51% with TnGtsf1, 58% with PiGtsf1, and 55% with OfGtsf1 (Figure S2). Phylogenetic analysis further confirmed that Gtsf1 is highly conserved across Lepidoptera, with PxGtsf1 clustering within the lepidopteran monophyletic group (Figure 1a).

Developmental expression profiling revealed that *PxGtsf1* exhibited stage-specific expression patterns across the *P. xylostella* life cycle, with the highest transcript levels in male adults and male pupae, and relatively low expression in early larval stages (Figure 1b). Tissue-specific expression analysis showed that *PxGtsf1* was predominantly expressed in the testes of male adults, with the lowest expression in the silk gland (Figure 1c). Collectively, these results suggest that PxGtsf1 may play a functional role in testis development and male reproductive processes in *P. xylostella*.

### 3.2. CRISPR/Cas9-Mediated Mutagenesis of PxGtsf1

**Figure 2:**
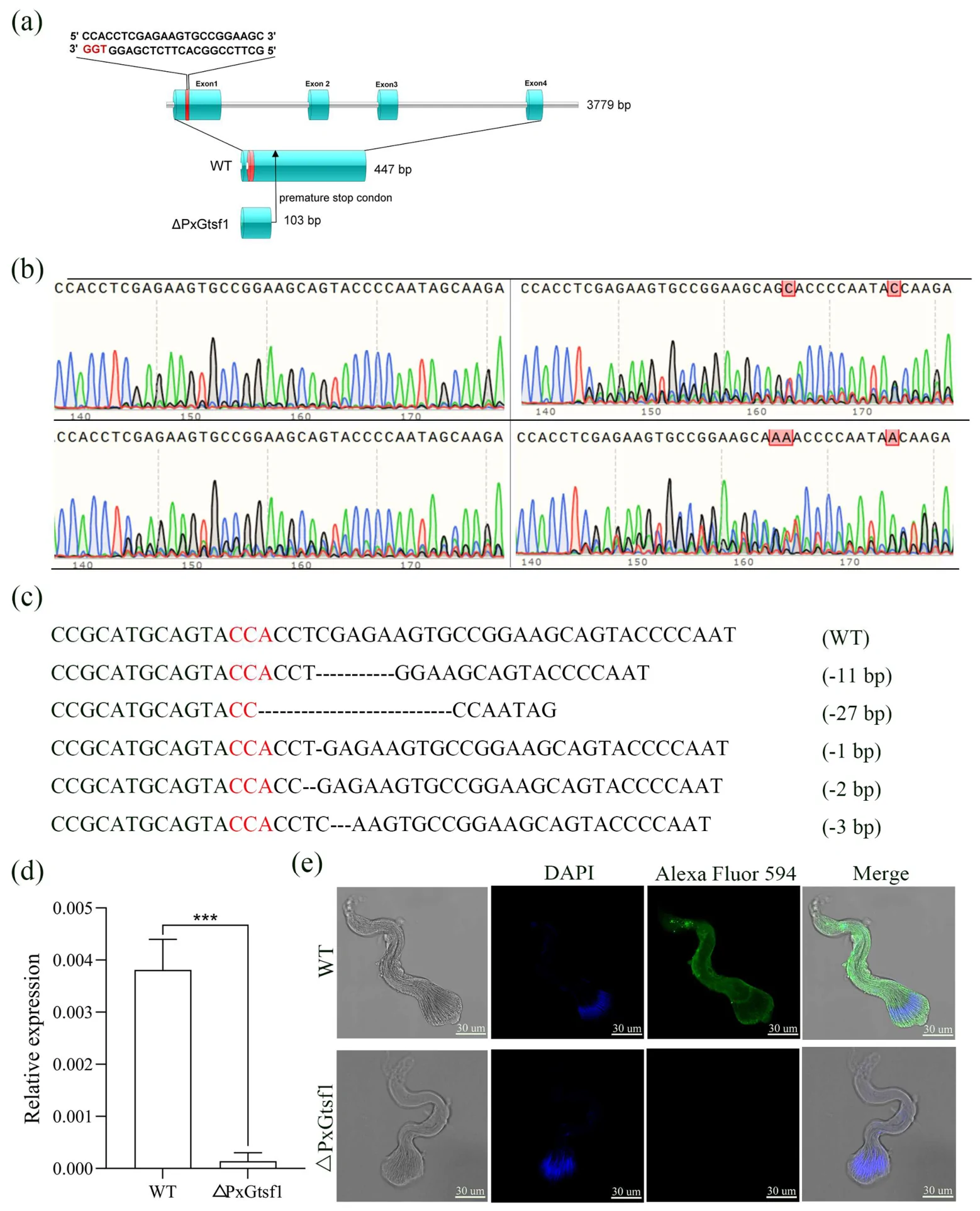
CRISPR/Cas9-mediated knockout of *PxGtsf1*. (**a**) Schematic representation of *PxGtsf1* gene structure, sgRNA target site and truncated *PxGtsf1*. The red line indicates the sgRNA target site, and black arrowhead denotes the premature stop codon. (**b**) Sanger sequencing chromatogram of G_0_ adults showing overlapping peaks at the target site. Colored peaks represent DNA bases: blue, cytosine (C); green, adenine (A); black, guanine (G); red, thymine (T). (**c**) Five distinct indel mutations in F_1_ individuals, including two in-frame deletions (−27 bp and −3 bp) and three frame-shift deletions (−1 bp, −2 bp and −11 bp). Red color bases indicate the PAM sequence, and dashed lines represent the deleted bases. (**d**) Relative *PxGtsf1* transcript levels in △PxGtsf1 and WT. (**e**) Immunofluorescence assay showing absence of detectable PxGtsf1 protein signal in ΔPxGtsf1 compared with WT. Nuclei are stained with DAPI (blue). PxGtsf1 protein is detected by specific primary antibody and Alexa Fluor 594-conjugated secondary antibody (pseudocolored green). Merged images show the overlay of DAPI and PxGtsf1 signals. Scale bar, 30 μm. Bars highlight the mean ± SE, and three asterisks (***) represent the highly significant difference with *p* < 0.001 between relative expressions of *PxGtsf1* in WT and mutant individuals.

A total of 200 preblastodermal *P. xylostella* embryos were microinjected with sgRNA-Cas9 ribonucleoprotein (RNP) complexes, resulting in an embryo hatching rate of 47.5% (95/200). Among the hatched larvae, 66.3% (63/95) successfully developed into adults and were defined as the G_0_ generation. Sanger sequencing analysis revealed overlapping peaks at the target site within exon 1 of the *PxGtsf1* gene (Figure 2a), a characteristic signature of CRISPR-induce mutations (Figure 2b). Sequencing of all G_0_ individuals confirmed an overall mutation efficiency of 22.2% (14/63).

In the F_1_ generation, five distinct deletion types were identified, including 11 bp, 27 bp, 1 bp, 2 bp, and 3 bp deletions (Figure 2c). Offspring carrying the 11 bp deletion were selected to establish the F_2_ generation for sib-crossing. Homozygous individuals were obtained in the F_3_ generation, and subsequent sib-crossed of these homozygotes yielded a stable *PxGtsf1* homozygous mutant line, hereafter referred to as ΔPxGtsf1 (Figure S3). The 11 bp deletion in exon 1 induced a frameshift mutation, introducing a premature stop codon 103 bp downstream of the mutation site (Figure 2a).

RT-qPCR analysis showed that *PxGtsf1* transcript levels were significantly downregulated in ΔPxGtsf1 compared with the WT (Figure 2d). Consistently, immunofluorescence assays detected no PxGtsf1 protein signal in the ΔPxGtsf1 individuals (Figure 2e). These findings confirm the successful generation of a homozygous *PxGtsf1* mutant line carrying an 11 bp deletion, which introduces a premature stop codon and results in complete loss of PxGtsf1 function at both transcript and protein levels.

### 3.3. PxGtsf1 Knockout Does Not Affect Female Sex Determination-Related Traits in P. xylostella

**Figure 3:**
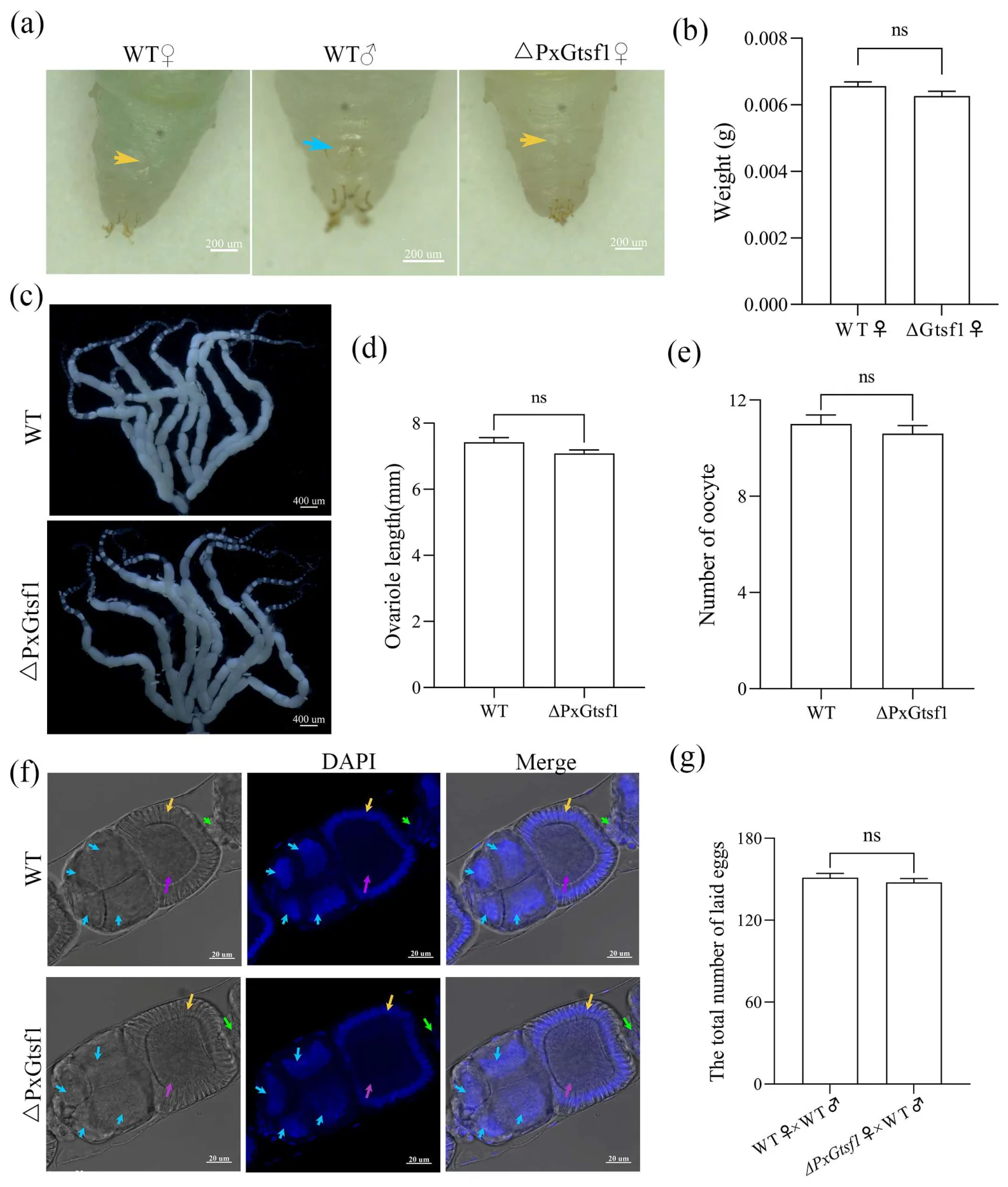
Effects of *PxGtsf1* knockout on female development of *P. xylostella*. (**a**) Morphological comparison of pupal external genitalia. WT female pupae exhibit a characteristic “X”-shaped structure at the abdominal gonopore (yellow arrows), whereas WT male pupae display paired protuberances at the gonopore of the ninth abdominal segment (blue arrows). ΔPxGtsf1 females show an identical “X”-shaped structure to WT females, indicating normal female morphology development. (**b**) Comparison of pupal weights between WT and ΔPxGtsf1 females. (**c**) Ovarian morphology of WT and ΔPxGtsf1 females, both displaying normal ovarian structures with fully developed ovarioles. (**d**) Quantification of ovariole length in WT and ΔPxGtsf1 females, with no significant difference detected between groups (*p* > 0.05). (**e**) Comparison of the number of mature oocytes between WT and ΔPxGtsf1 females. (**f**) Confocal images of ovarioles stained with DAPI (blue). Blue arrowheads indicate nurse cells, yellow arrowheads indicate follicle cells, purple arrowheads indicate oocytes, and green arrowheads indicate stalk cells. No obvious morphological abnormalities were observed in ΔPxGtsf1 ovaries compared to WT. (**g**) Total number of eggs laid by different mating combinations over three days. Data are presented as mean ± SE; “ns” indicates no significant difference (*p* > 0.05).

To determine whether the *PxGtsf1* gene regulates female-specific traits in *P. xylostella*, we conducted morphological examinations of the external genitalia and ovaries in ΔPxGtsf1 and WT female pupae. Both groups displayed the characteristic “X”-shaped external genitalia structure, with no signs of masculinization (Figure 3a). Additionally, pupal weights did not differ significantly between ΔPxGtsf1 and WT females (Figure 3b). Ovarian morphology was also unaffected by *PxGtsf1* knockout, with both ΔPxGtsf1 and WT females possessing eight well-developed ovarioles (Figure 3c). Ovariole length showed no significant differences between the two groups (*t* = 1.969, *df* = 26, *p* = 0.0597; Figure 3d). DAPI staining revealed the normal organization of oocytes, nurse cells, follicle cells, and stalk cells in ΔPxGtsf1 ovaries (Figure 3e). Furthermore, the number of fully matured oocytes in newly emerged females showed no significant difference between ΔPxGtsf1 and WT individuals (*t* = 0.7917, *df* = 28, *p* = 0.6596; Figure 3f). Compared with the cross of WT males mated with WT females, the total 3-day egg production did not differ significantly in the cross of ΔPxGtsf1 females mated with WT males (*t* = 0.8379, *df* = 38, *p* = 0.4074; Figure 3g). Consistently, no significant difference in egg hatching rate was detected in all cross combinations (*t* = 1.343, *df* = 46, *p* = 0.1858; Figure S4a). These results collectively demonstrate that loss of PxGtsf1 function does not impair female morphological traits, oogenesis, or fertility.

Expression profiling of sex-related genes further validated these phenotypic observations. Transcript levels of *PxFem-1A*, *PxFem-1B*, *PxFem-1C* (Figure S4b–d), *PxMasc* (a downstream target of the *Fem* piRNA pathway; Figure S4e), *PxIMP* (IGF-II mRNA-binding protein; Figure S4f), *PxPSI* (P-element somatic inhibitor; Figure S4g), *PxVg* (vitellogenin; Figure S4h), and *PBP1* (pheromone-binding protein 1; Figure S4i) were comparable between ΔPxGtsf1 mutants and WT adults. Taken together, our findings demonstrate that PxGtsf1 is dispensable for female sex determination and reproductive development in *P. xylostella*.

### 3.4. PxGtsf1 Knockout Results in Reduced Male Fertility in P. xylostella

**Figure 4:**
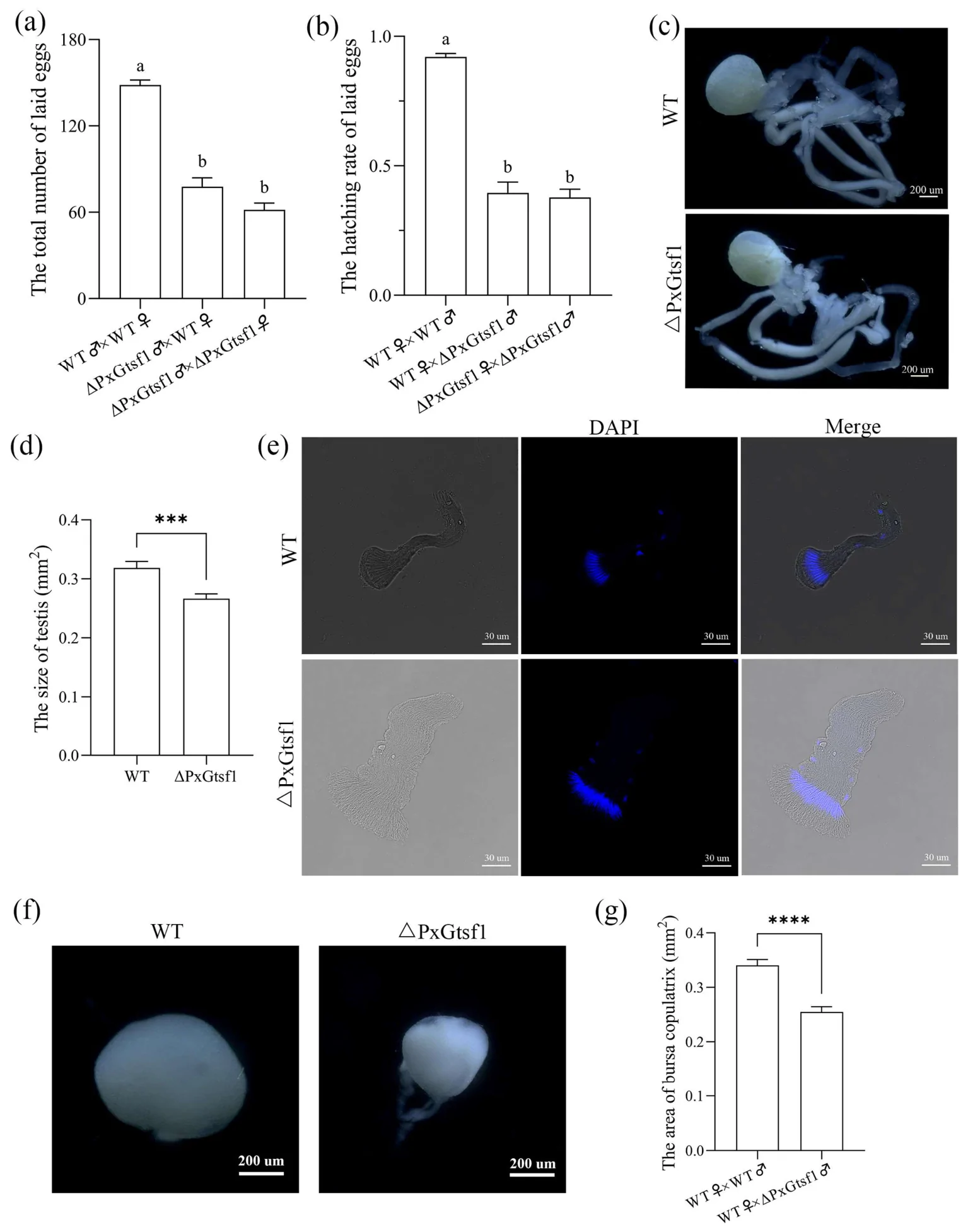
Effects of *PxGtsf1* knockout on male reproductive and associated traits in *P. xylostella*. (**a**) Total number of eggs laid by different mating combinations over three days. (**b**) The hatching rate over a 3-day period across different mating combinations. (**c**) Morphology of male reproductive tissues in WT and ΔPxGtsf1 adults, showing comparable gross structural organization. (**d**) Comparison of testis size between WT and ΔPxGtsf1 males. (**e**) Morphological comparison of eupyrene sperm bundles in WT and ΔPxGtsf1 males. Blue fluorescence indicates DAPI-stained cell nuclei. (**f**) Morphological comparison of bursa copulatrix across different mating combinations. (**g**) Comparison of bursa copulatrix distention area in females mated with WT versus ΔPxGtsf1 males. The data are shown as mean ± SE. Lowercase letters indicate statistical differences based on multiple-comparison test. Bars sharing identical letters are not significantly different from each other, whereas bars with different letters differ significantly (*p* < 0.05). The three asterisks (***) represent a highly significant difference with *p* < 0.001. The four asterisks (****) represent a highly significant difference with *p* < 0.0001.

Compared to control pairings with WT males, the total number of eggs laid over a 3-day period was significantly reduced when ΔPxGtsf1 males were mated with either WT or ΔPxGtsf1 females (*F* = 83.19, *df*_1_ = 2, *df*_2_ = 87, *p* < 0.001; Figure 4a). Consistently, egg hatching rates were markedly lower in crosses involving ΔPxGtsf1 males than those with WT males (*F* = 98.11, *df*_1_ = 2, *df*_2_ = 84, *p* < 0.001; Figure 4b). These findings indicate that *PxGtsf1* knockout severely impairs male reproductive capacity but does not compromise female fecundity in *P. xylostella*.

Morphological examination of male reproductive tissues revealed that the gross structure of ΔPxGtsf1 testes was similar to that of WT controls (Figure 4c). However, quantitative analysis demonstrated that the average testis area was significantly reduced in ΔPxGtsf1 males (Figure 4d), suggesting impaired testicular development. Subsequent DAPI staining and fluorescent microscopy analysis further showed that mature apyrene sperm bundles in ΔPxGtsf1 males exhibited normal morphology with dispersed nuclei, similar to those in WT males (Figure S5a). In contrast, eupyrene sperm bundles in ΔPxGtsf1 males displayed abnormal swelling despite maintaining normal nuclear localization (Figure 4e), indicating that PxGtsf1 knockout selectively disrupts eupyrene sperm morphogenesis.

WT females were paired with either WT or ΔPxGtsf1 males for a 40 min trial period. The bursa copulatrix distention area dissected from females mated with ΔPxGtsf1 males was significantly smaller than that of females mated with WT males (Figure 4f,g). DAPI staining and microscopic observation confirmed the presence of both apyrene and eupyrene sperm in the bursa copulatrix, with no notable differences in nuclear morphology between groups (Figure S5b).

Furthermore, *PxGtsf1* knockout had no discernible effects on mating behavior, as no significant differences were detected in response time (*F* = 1.955, *df*_1_ = 2, *df*_2_ = 70, *p* = 0.1492; Figure S6a) and mating duration (*F* = 1.114, *df*_1_ = 2, *df*_2_ = 55, *p* = 0.3355; Figure S6b) across different mating combinations. Collectively, these results demonstrate that PxGtsf1 is essential for proper testicular development and eupyrene sperm integrity, but not for mating behavior in males *P. xylostella*.

### 3.5. PxGtsf1 Physically Interacts with PxPiwi

**Figure 5:**
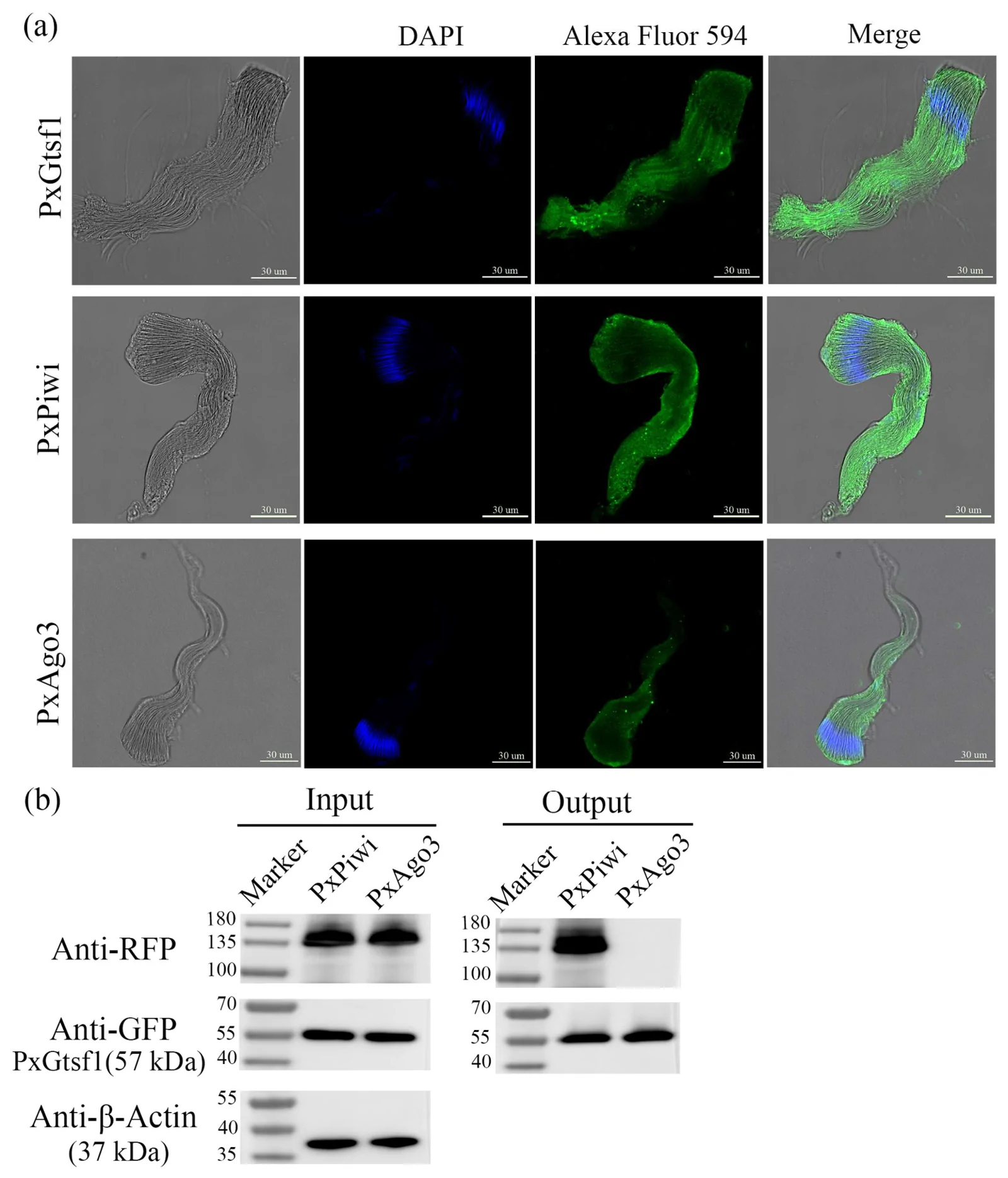
Interaction between PxGtsf1 and PxPiwi in *P. xylostella*. (**a**) Subcellular localization of PxGtsf1, PxPiwi and PxAgo3 in the sperm bundle. Nuclei were counter-stained with DAPI (blue). PxGtsf1, PxPiwi and PxAgo3 proteins were each detected using corresponding specific primary antibodies and Alexa Fluor 594-conjugated secondary antibodies (pseudocolored green). Merged images show the overlay of DAPI nuclear signal and target-protein fluorescence. Scale bar, 30 μm. (**b**) Co-IP assay demonstrating physical interaction between PxGtsf1 and PxPiwi. Western blot analysis shows that PxPiwi was co-precipitated with PxGtsf1, while no interaction was detected between PxGtsf1 and PxAgo3.

Immunofluorescence analysis revealed that PxGtsf1, PxPiwi, and PxAgo3 were all localized within sperm bundles, and their fluorescence signals exhibited nearly complete colocalization (Figure 5a). To investigate whether PxGtsf1 directly interacts with PxPiwi and PxAgo3, we generated recombinant fusion proteins: PxGtsf1-RFP, PxPiwi-GFP, and PxAgo3-GFP. Co-IP assays demonstrated that PxGtsf1 specifically interacted with PxPiwi, but no interaction was detected between PxGtsf1 and PxAgo3 (Figure 5b).

### 3.6. PxGtsf1 Knockout Disrupts Transposon Homeostasis in P. xylostella

**Figure 6:**
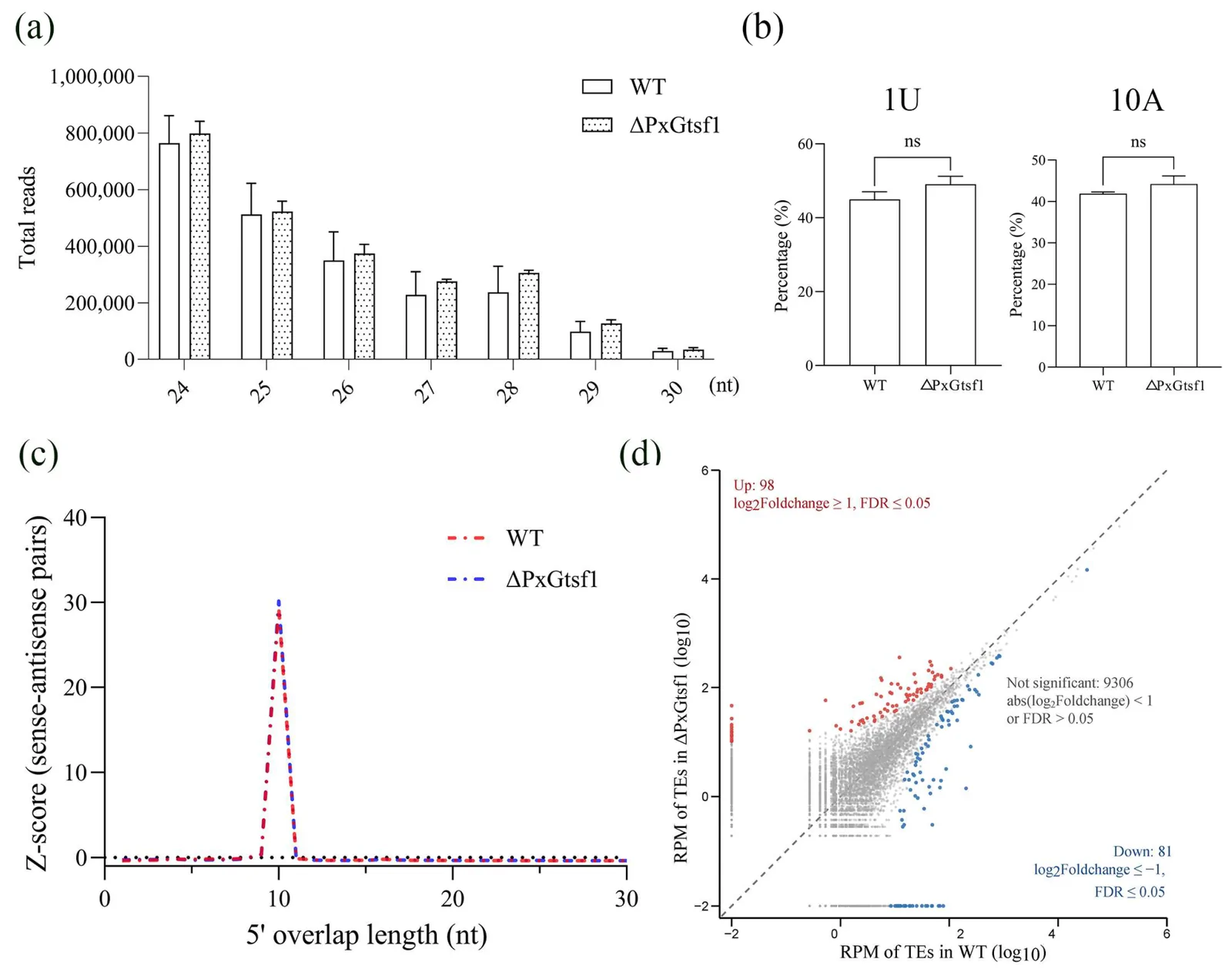
Effects of *PxGtsf1* knockout on piRNA profiles and transposon expression in *P. xylostella* testes. (**a**) Length distribution and relative abundance of small RNAs in testes from WT and ΔPxGtsf1 males. (**b**) Sequence logos display conserved 1U/10A ping-pong signatures in two genotypes. “ns” indicates no significant difference (*p* > 0.05). (**c**) Overlap frequency distribution of sense–antisense piRNA pairs, both groups show a prominent 10 nt overlap peak. (**d**) Scatterplot of TE expression (RPM normalization) between WT and ΔPxGtsf1. Dots in gray represent all transposable-element loci. Red dots indicate significantly up-regulated TE loci, and blue dots represent significantly down-regulated TE loci upon *PxGtsf1* knockout. The diagonal black line indicates equal expression levels.

To investigate whether PxGtsf1 is involved in piRNA maturation and transposon silencing, we performed small RNA sequencing on testes dissected from WT and ΔPxGtsf1 mutant males. Examination of piRNA abundance and length distribution profiles showed no significant differences between WT and ΔPxGtsf1 testes (Figure 6a). We next evaluated the conserved molecular signatures associated with the ping-pong amplification cycle using multi-mapping piRNA reads. Analysis of nucleotide composition revealed strong 1U enrichment at the 5′ end of antisense piRNAs and 10A enrichment at the 10th nucleotide position of sense piRNAs in both WT and ΔPxGtsf1 samples. No significant differences were observed in either 1U or 10A enrichment between WT and ΔPxGtsf1 libraries, indicating that the nucleotide preferences associated with primary piRNA loading and secondary piRNA amplification were largely maintained after *PxGtsf1* knockout (Figure 6b). Furthermore, genome-wide analysis of sense–antisense piRNA interactions showed a characteristic 10-nt 5′–5′ overlap peak in both WT and ΔPxGtsf1 libraries (Figure 6c), confirming the presence of an active ping-pong amplification signature. Together, these results indicate that loss of PxGtsf1 does not significantly affect the global piRNA amplification cycle or the conserved molecular features of piRNA processing. To visualize global TE expression variation, TE expression levels normalized to RPM were plotted as scatter plots for pairwise comparison. A total of 179 differentially regulated TEs were identified between WT and ΔPxGtsf1 testes, including 98 upregulated and 81 downregulated TEs (Figure 6d). The altered TE expression profiles in ΔPxGtsf1 testes indicate that *PxGtsf1* contributes to maintaining TE homeostasis, although the overall piRNA abundance and conserved ping-pong signatures remain largely unchanged. These findings suggest that *PxGtsf1* is not required for global piRNA abundance or ping-pong amplification but plays a specific role in regulating TE activity in the testes of *P. xylostella*.

## 4. Discussion

Gtsf1, functioning as a core cofactor of the piRNA pathway, exhibits remarkable functional evolutionary plasticity across species ranging from unicellular protozoan to mammals [5,7,9,11,12,13,24]. In the present study, we systematically investigated the biological functions of PxGtsf1 using CRISPR/Cas9 mediated gene knockout, and revealed fundamental functional divergence between PxGtsf1 and its silkworm ortholog BmGtsf1, providing direct evidence for the functional diversification of Gtsf1 within Lepidoptera.

We first confirmed the high conservation of PxGtsf1 among lepidopteran insects through sequence alignment and phylogenetic analysis. PxGtsf1 contains two canonical ZF U11 48K domains, consistent with the structural features of Gtsf1 proteins reported in mouse, *Drosophila*, and silkworm [2,6,13]. Developmental and tissue specific expression profiling revealed that *PxGtsf1* is predominantly expressed in testes, with peak expression levels in male adults. This expression pattern shares both similarities with and differences from the expression profiles in *Drosophila* and silkworm [11,13], suggesting that PxGtsf1 may primarily participate in male reproductive processes rather than female sex determination.

In striking contrast to the partial female to male sex reversal caused by *BmGtsf1* mutations [13], *PxGtsf1* knockout in the present study exerted no significant effects on female external genital morphology, ovarian development, oogenesis, or fecundity. Moreover, the expression of key sex determination pathway genes remained unaltered between WT and mutant individuals. While female sex determination in the silkworm depends on W chromosome-derived Fem piRNA, which suppresses the masculinization factor BmMasc through the BmGtsf1-BmSiwi complex [14,15], the sex determination system of *P. xylostella* involves W-linked *PxyMasc* retrocopies and does not rely on a *Fem* piRNA pathway [17]. Our findings, together with previous studies, suggest that the ancestral role of Gtsf1 in germline protection, transposon regulation, and male reproductive development is conserved between *B. mori* and *P. xylostella*. The female sex-determination function of BmGtsf1 likely represents a lineage-specific co-option of a conserved Gtsf1-mediated regulatory module into the *Fem* piRNA-based sex-determination cascade of the silkworm rather than an intrinsic functional divergence of the Gtsf1 protein itself In *P. xylostella*, where sex determination is mediated by *Masc* retrocopy-associated regulation rather than a *Fem* piRNA pathway, *PxGtsf1* does not appear to participate in female sex determination and instead maintains conserved functions associated with germline genome stability, transposon regulation, and male reproductive development. We propose that the recruitment of Gtsf1 into female sex-determination pathways may have occurred through lineage-specific adaptation of piRNA-mediated regulatory networks. Thus, the functional differences observed between BmGtsf1 and PxGtsf1 likely reflect differential utilization of a conserved germline regulatory factor in distinct evolutionary contexts rather than a loss or divergence of its ancestral role.

In terms of male reproduction, *PxGtsf1* knockout resulted in reduced testis area and aberrant eupyrene sperm bundle morphology, ultimately leading to a significant decline in male fertility. This phenotype resembles mouse *Gtsf1* deficiency induced male sterility in terms of reproductive endpoints, but the underlying mechanisms appear to differ. Mouse *Gtsf1* primarily affects secondary piRNA biogenesis, leading to transposon derepressing and meiotic arrest [6]. In contrast, although *PxGtsf1* deficiency caused dysregulated transposon expression, overall piRNA abundance remained largely unchanged. This observation suggests that *PxGtsf1* may influence testicular and sperm development primarily through regulating transposon activity rather than affecting overall piRNA production. This finding aligns with the role of DmGtsf1 as a core component of the Piwi mediated transcriptional silencing complex without participating in piRNA biogenesis [11], but contrasts with the essential role of mouse Gtsf1 in secondary piRNA biogenesis [6].

Notably, co-immunoprecipitation assays confirmed an interaction between PxGtsf1 and PxPiwi, but not with PxAgo3, consistent with the finding that BmGtsf1 interacts specifically with BmSIWI [15]. Piwi proteins are core effectors of the piRNA pathway, mediating transposon silencing in germ cells to safeguard genomic integrity [25]. Structural studies have revealed that Gtsf1 specifically recognizes the duplex formed between a piRNA and its target RNA, thereby stabilizing Siwi in a catalytically active conformation and enhancing the efficiency of target RNA cleavage [26]. The PIWI complex, comprising PIWI, Gtsf1, and Maelstrom, is evolutionarily conserved across the animal kingdom, with similar assembly mechanisms found from sponges to humans [3,26]. The confirmation of the PxGtsf1-PxPiwi interaction in the lepidopteran pest *P. xylostella* provides further evidence for the evolutionary conservation of this regulatory mechanism. Although PxGtsf1 and PxAgo3 co-localized within sperm bundles, no direct protein–protein interaction was detected. Ago3 is a key component of the piRNA “ping-pong” amplification cycle, primarily responsible for carrying sense-strand piRNAs and cleaving antisense transcripts [27]. This result suggests that PxGtsf1 may selectively regulate the Piwi branch of the pathway, with a lesser role in the Ago3-mediated amplification loop. This functional specificity is consistent with findings in *Drosophila*, where the Gtsf1 ortholog, Tpp, primarily influences the piRNA loading and germ plasm localization of Aubergine (Aub, a Piwi family member), with limited effects on Ago3 function [28], similar observations have also been made in the silkworm [15]. Small RNA sequencing revealed that a substantial number of transposons were differentially expressed in the absence of significant changes to the overall piRNA pool, indicating that PxGtsf1 likely functions at the level of transposon epigenetic silencing rather than piRNA biogenesis. It is noteworthy that the function of Gtsf1 family members exhibits some inter-species variation: loss of *DmGtsf1* in *Drosophila* does not impact piRNA synthesis [12]. whereas *Gtsf1* knockout results in reduced piRNA levels in mice and silkworms [6,13]. Notably, *PxGtsf1* knockout resulted in bidirectional changes in transposon expression within the testis. Studies have shown that Gtsf1 can preferentially regulate specific classes of transposons through its tRNA-binding property, and its loss leads to the specific upregulation of these transposon classes [29]. Concurrently, Gtsf1 deficiency may disrupt the piRNA screening and degradation processes, resulting in aberrant accumulation of non-transposon-derived piRNAs, which in turn alters the composition and targeting specificity of the piRNA repertoire, causing certain transposons to be misrecognized and consequently downregulated [9]. Furthermore, as Gtsf1 functions as a catalytic enhancer for PIWI proteins, differential sensitivity of various transposons to PIWI cleavage efficiency may also contribute to their distinct responses to Gtsf1 loss [30]. The downregulation of some transposons might additionally reflect compensatory silencing mechanisms activated by cells in response to transposon derepression [2]. Therefore, the bidirectional changes in transposon expression induced by *PxGtsf1* knockout underscore the multifaceted roles of Gtsf1 within the piRNA pathway and the inherent complexity of the transposon regulatory network. This mechanism is consistent with the phenotypes observed in our study, including reduced testis size and abnormal development of eupyrene sperm, suggesting a potential causal relationship. Derepressed transposons may undergo de novo insertional mutagenesis, thereby disrupting the function of essential genes. This mechanistic divergence further expands our understanding of the functional diversity of Gtsf1.

## 5. Conclusions

In summary, this study provides the first characterization of the biological functions of PxGtsf1 in *P. xylostella*, demonstrating that Gtsf1 functions are not uniformly conserved within Lepidoptera but exhibit notable species specificity. PxGtsf1, as a testis enriched factor, safeguards proper testicular development and eupyrene sperm morphogenesis through its interaction with PxPiwi and maintenance of transposon homeostasis, yet plays no role in female sex determination. From an evolutionary perspective, although both the silkworm and *P. xylostella* belong to Lepidoptera, Gtsf1 has undergone significant functional divergence between these two species, a divergence that may be associated with differences in their upstream sex determining signaling pathways or driven by differential transposon landscapes across species. This study not only enriches our understanding of the functional evolutionary plasticity of Gtsf1 but also provides a potential novel target for genetic pest management strategies based on male sterility in lepidopteran pests.

## Data Availability

The original contributions presented in this study are included in the article. Further inquiries can be directed to the corresponding author.

## Supplementary Materials

The following supporting information can be downloaded at: https://www.mdpi.com/article/10.3390/insects17080846/s1, Table S1: Primers used in this study; Figure S1: The conserve domains of Gtsf1; Figure S2: Multiple alignment of amino acid sequence of Gtsf1 in lepidopteran species including *B. mori* (*Bm*), *L. glycinivorella* (*Lg*), *S. frugiperda* (*Sf*), *T. ni* (*Tn*), *P. interpunctella* (*Pi*), and *O. furnacalis* (*Of*); Figure S3: The sequence chromatogram for 11-bp deletion homozygous individuals; Figure S4: Effects of *PxGtsf1* knockout on hatching rate and the expressions of female sex determination genes in *P. xylostella*; Figure S5: Effects of *PxGtsf1* knockout on apyrene sperm bundle and sperms in bursa copulatrix of *P. xylostella*; Figure S6: Effects of *PxGtsf1* knockout on response time and mating duration of *P. xylostella*.
