# Supplementary material for: Gametocyte-Specific Factor 1 Is Essential for Male Fertility and Transposon Homeostasis in Plutella xylostella
Source: Insects. 2026 Aug 14;17(8):846. doi: 10.3390/insects17080846 (PMC13513780; doi:10.3390/insects17080846)
Supplement: Supplementary file 1 [file insects-17-00846-s001.zip › insects-4451126-supplementary.pdf]

Supplementary Information

Table S1 Primers used in this study

Primer name	Nucleotide sequence (5’-3’)	Use
PxGtsf1-F	GTACCACCTCGAGAAGTGCC	Gene cloning
PxGtsf1-R	GCCGGTAGTTCTTGACTCCC	
PxGtsf1-qPCR-F	CCCAATAGCAAGAAAGTCAGC	RT-qPCR
PxGtsf1-qPCR-R	CAATCGTCAACGGCGAAGC	
RPL32-qPCR-F	CAATCAGGCCAATTTACCGC	
RPL32-qPCR-R	CTGGGTTTACGCCAGTTACG	
SgRNA F	TAATACGACTCACTATAGGGCTTCCGGCACTTCTCGAGGGTTTTAGAGCTAGAAATAGCAAGTTAAAATAAGGCTAGTCC	SgRNA synthesis
SgRNA R	AAAAGCACCGACTCGGTGCCACTTTTTCAAGTTGATAACGGACTAGCCTTATTTTAACTTGCTATTTCTAGCTCTAAAAC	
PxGtsf1-seq-F	CAGCGCAATAACCAACCAG	Mutation detection
PxGtsf1-seq-R	CAGCCAAAATGTGAGTAGCG	
PxMasc-qPCR-F	CCCAATAGCAAGAAAGTCAGC	Sex determina-tion gene checking
PxMasc-qPCR-R	CAATCGTCAACGGCGAAGC	
Pxdsx-qPCR-F	CAATCAGGCCAATTTACCGC	
Pxdsx-qPCR-R	CTGGGTTTACGCCAGTTACG	
PxPBP1-qPCR-F	TCGGTAAAGCTTTGGGTGAC	
PxPBP1-qPCR-R	GTTTAAGCGCGAAGTCCTTG	
Pxvg-qPCR-F	GCTTCTGGTACACGGAGGAG	
Pxvg-qPCR-R	GACCATGCTGGTGTACATGG	

PxIMP-qPCR-F	CGTCCATCAACGACATCAGCA
PxIMP-qPCR-R	ATCATCGCCATCGGGTGC
PxPSI-qPCR-F	CAAGAGCGGCAAGACCATCA
PxPSI-qPCR-R	CGGCCAACAGTTCAAACACC
PxFem-1A-F	GACTCATAGTACCCCGCTGC
PxFem-1A-R	CCCTTGTAGCAGGCGATCAT
PxFem-1B-F	GCTCAGAAGAGGTGCAGAG
PxFem-1B-R	AGACACCAGATACAAGGCCG
PxFem-1C-F	ACTTGGAGACATGCTACTGC
PxFem-1C-R	CGCATCTAACTGCTCGTGTG

Note: The single underscore sequence represents the T7 promoter sequence, and the double underscore sequence represents the sgRNA scaffold sequence.

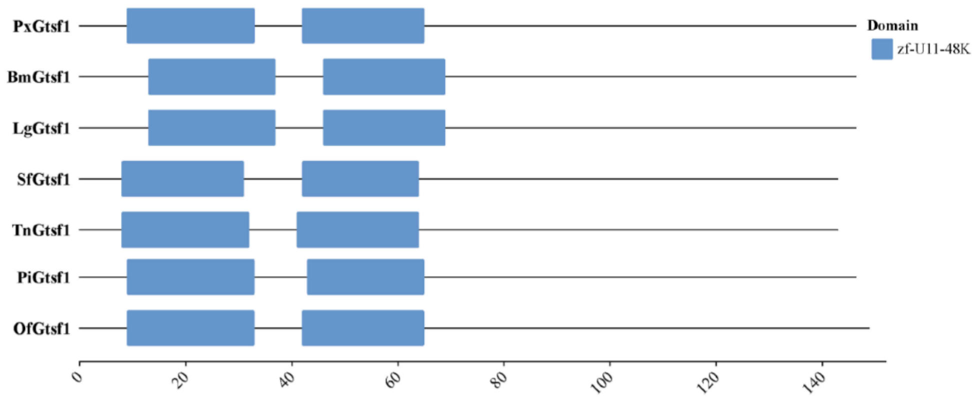

Figure S1　The conserve domains of Gtsf1.

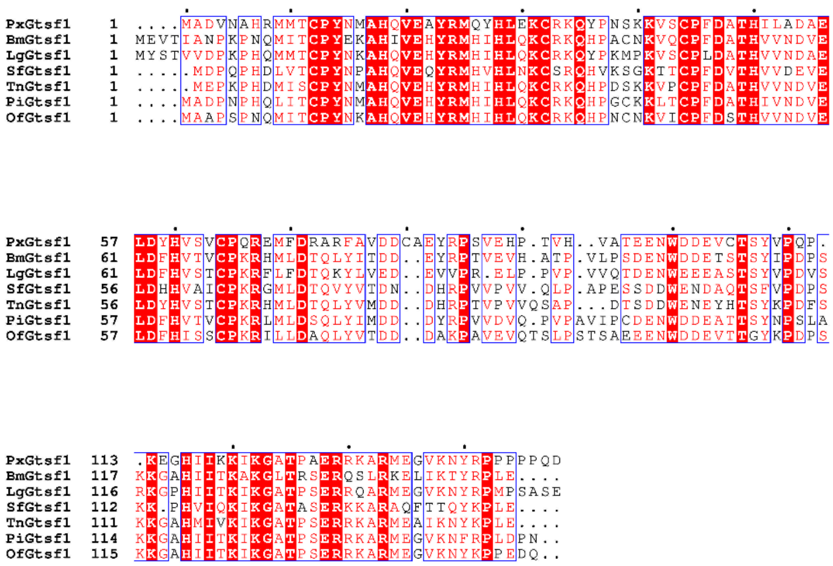

Figure S2 Multiple alignment of amino acid sequence of Gtsf1 in lepidopteran species including *B. mori* (*Bm*), *L. glycinivorella* (*Lg*), *S. frugiperda* (*Sf*), *T. ni* (*Tn*), *P. interpunctella* (*Pi*), and *O. furnacalis* (*Of*).

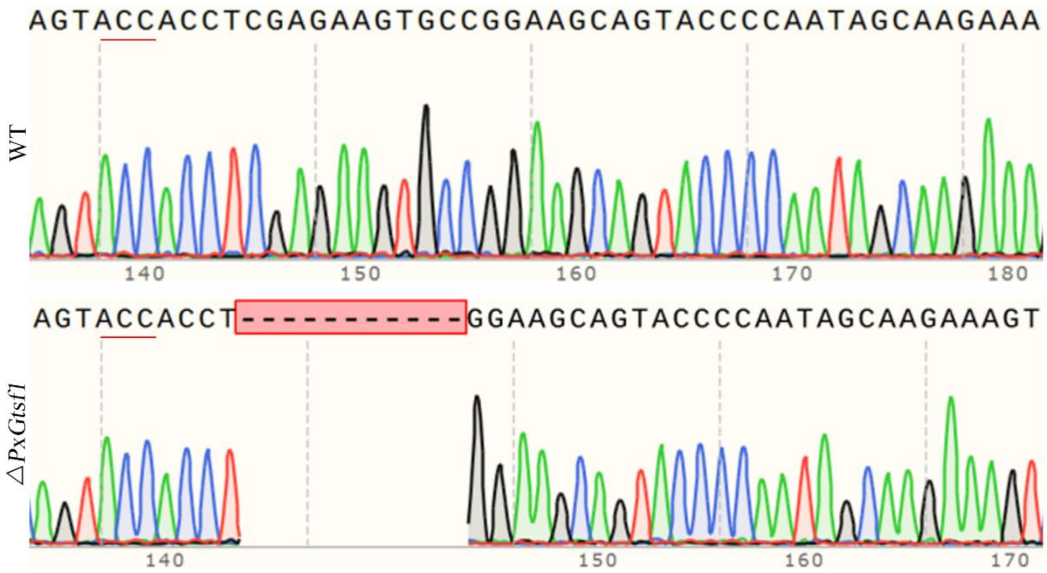

Figure S3 The sequence chromatogram for 11-bp deletion homozygous individuals. The red line indicates PAM.

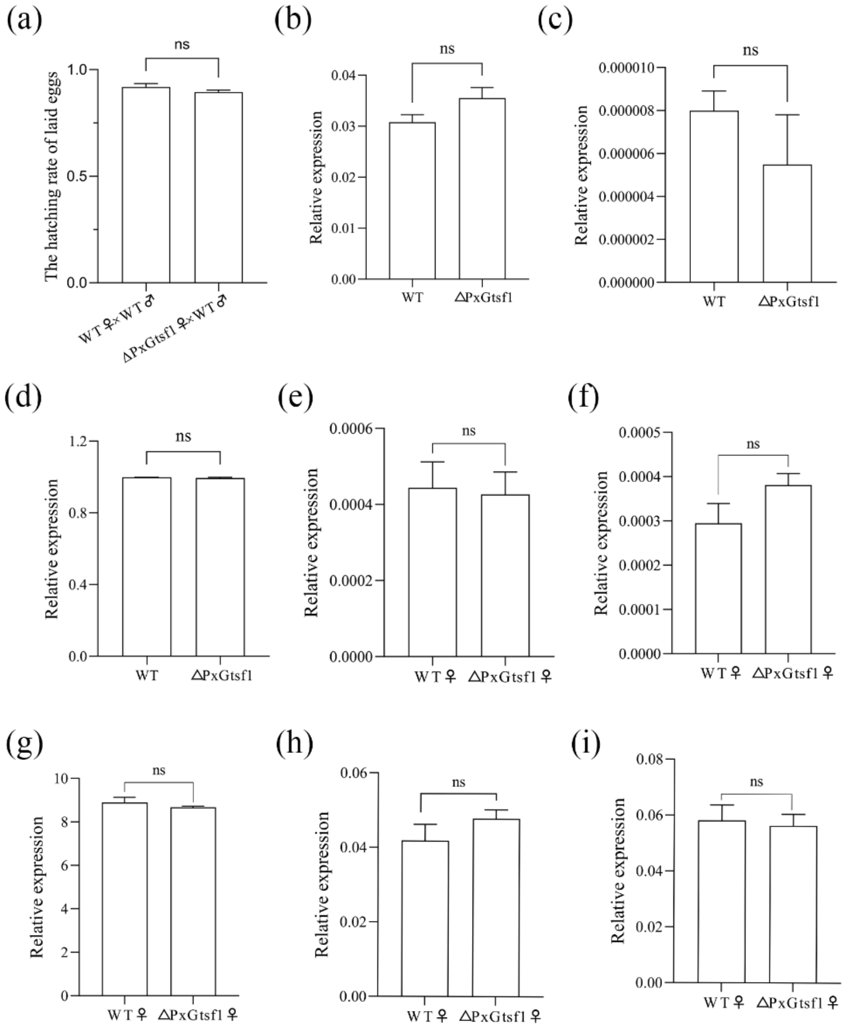

Figure S4 Effects of *PxGtsf1* knockout on hatching rate and the expressions of female sex determination genes in *P. xylostella.* (a) The hatching rate in different mating combinations. (b) The relative expression of *PxFem-1A* gene. (c) The relative expression of *PxFem-1B* gene. (d) The relative expression of *PxFem-1C* gene. (e) The relative expression of *PxMasc* gene. (f) The relative expression of *PxIMP* gene. (g) The relative expression of *PxPSI* gene. (h) The relative expression of *PxPBP1* gene. (i) The relative expression of *PxVg* gene.

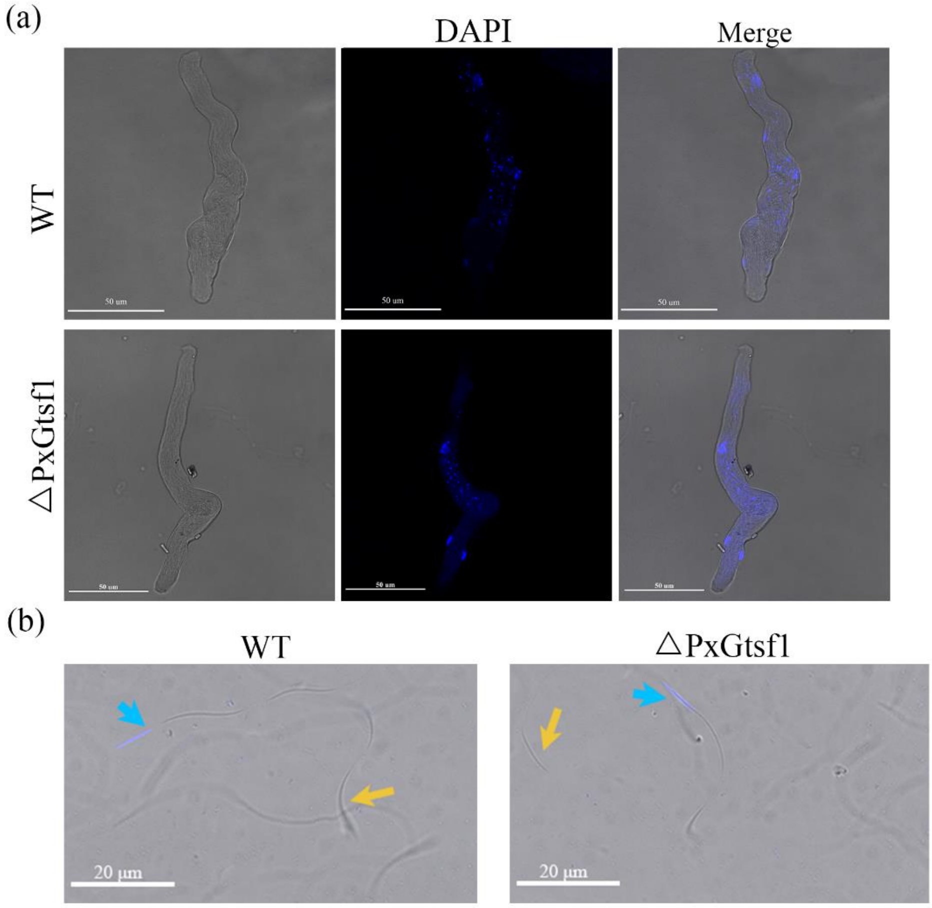

Figure S5 Effects of *PxGtsf1* knockout on apyrene sperm bundle and sperms in bursa copulatrix of *P. xylostella*. (a) Apyrene sperm bundles in WT and *PxGtsf1* mutant. Blue is the signal of DAPI for cell nucleus. (b) The morphology of sperm in bursa copulatrix. The blue arrowhead indicates eupyrene sperm, the yellow arrowhead indicates apyrene sperm. Blue is the signal of DAPI for cell nucleus.

(a)

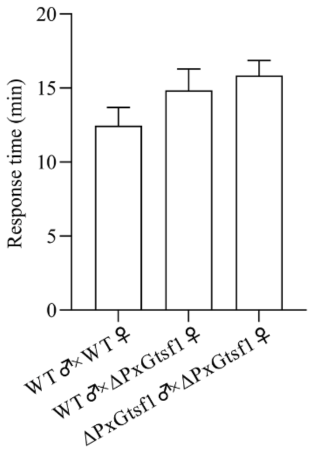

(b)

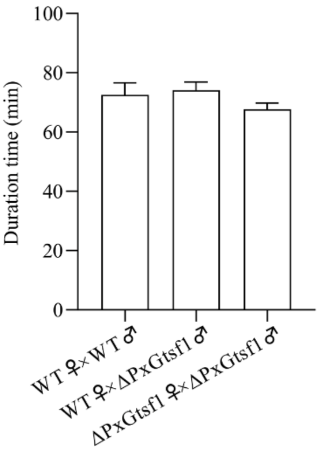

Figure S6 Effects of *PxGtsf1* knockout on response time and mating duration of *P. xylostella*. (a) Response time in crosses of different genotypes. (b) Duration time of different mating types. The data were shown as mean ± SE.
